# Supplementary material for: PURE-seq integrates FACS and PIP-seq for single-cell genomics of ultra-rare cells
Source: Nat Commun. 2026 Jan 21;17:1408. doi: 10.1038/s41467-025-68146-w (PMC12881479; doi:10.1038/s41467-025-68146-w)
Supplement: Supplementary file 1 — Supplementary Information [file 41467_2025_68146_MOESM1_ESM.pdf]

# Supplementary Figures:

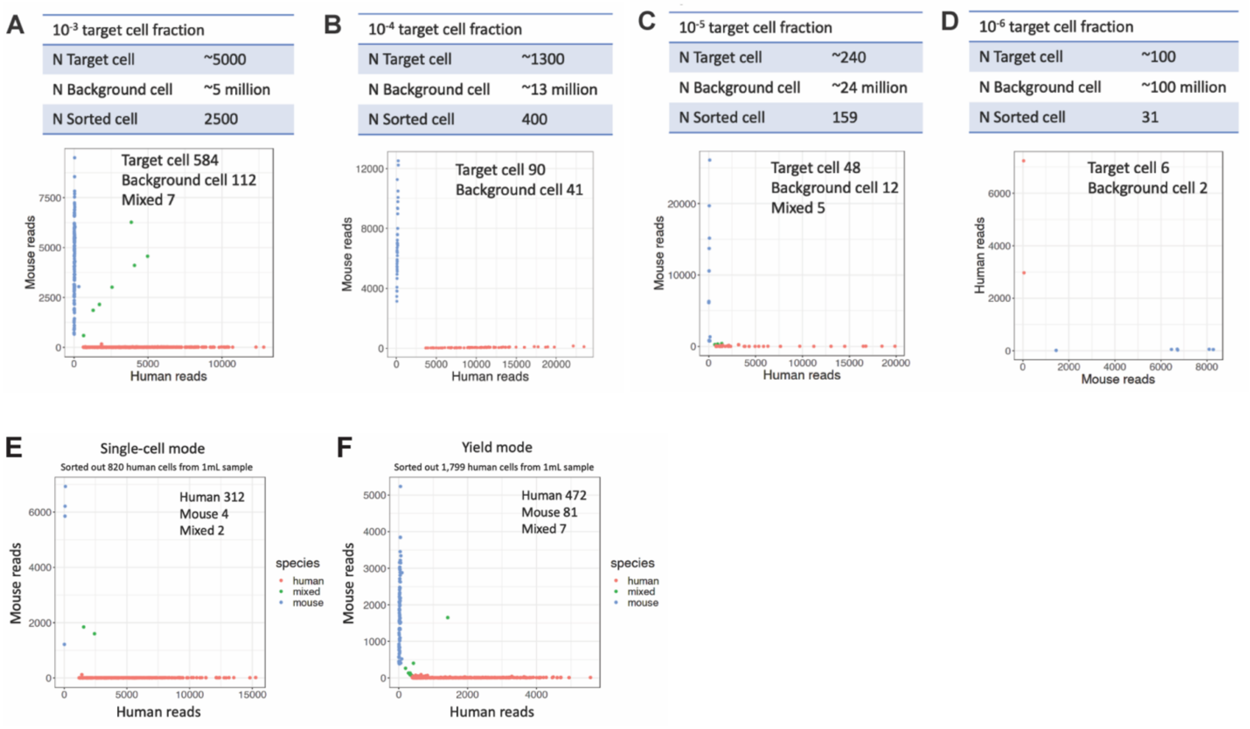


**Figure S1. Barnyard plots of $10^{-3}$, $10^{-4}$, $10^{-5}$ and $10^{-6}$ target cell fractions after sorting.** In each table, cell numbers for the corresponding dilution experiment sample are shown (N Target cell and N Background cell) and the number of sorted cells reported by FACS software is noted (N Sorted cell). In each barnyard plot, cells are colored by cell type (blue, mouse reads; red, human reads; green, mixed reads). **A-C)** Human HEK 293T cells and mouse NIH 3T3 cells were stained with Calcein Red-Orange and Calcein Green, respectively. Calcein Red-Orange-positive HEK 293T cells were sorted into PIPseq tubes. **D)** Mouse NIH 3T3 cells and human PBMCs were stained with Calcein Red-Orange and Calcein Green, respectively. Calcein Red-Orange-positive NIH 3T3 cells were sorted out as target cells. **E-F)** Recovery rate comparison of single-cell and yield sorting precision modes of FACS. Target cell fraction was $10^{-3}$ and the sample volume was controlled at 1mL. Compared with single-cell mode, yield mode sorted out 2-fold the number of total cells, and sequenced 1.5-fold the number of target rare cells from identical spike-in samples. The purities of single-cell and yield modes were 98% and 84%, respectively.

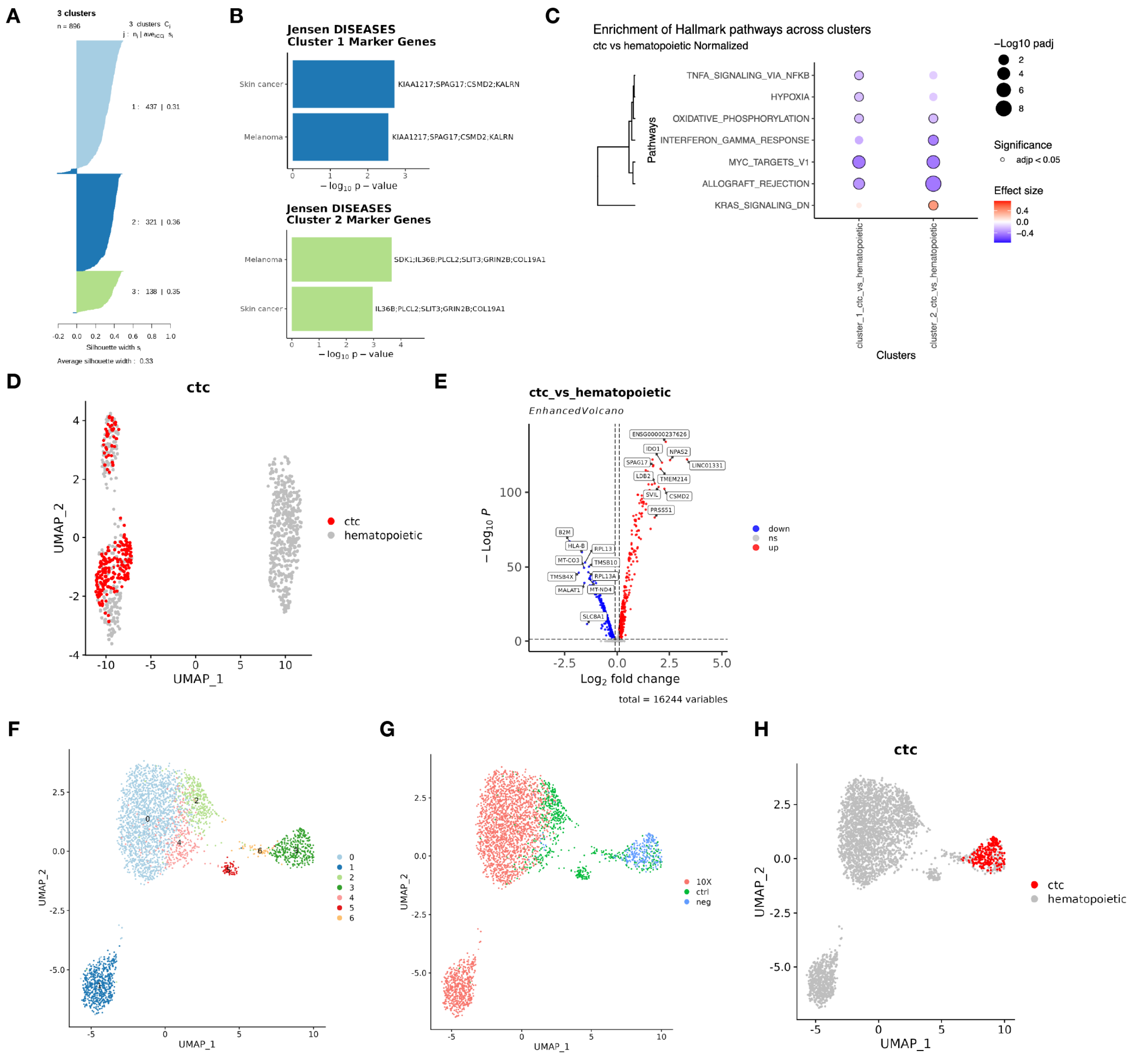


**Figure S2. Recovery rate comparison of single-cell and yield sorting precision modes of FACS. A)** Silhouette analysis reveals 3 clusters across 896 cells leads to robust cell type classification. **B)** Top disease types in the Jensen Diseases category of Enrichr are melanoma and skin cancer. **C)** Gene set enrichment analysis using the HALLMARK gene set of DEG comparisons between CTCs and hematopoietic cells in each CTC-enriched cluster. (red: high normalized enrichment score, blue: low normalized enrichment score) **D)** CTCs identified are highlighted in red. **E)** Differentially expressed genes comparing CTCs vs. hematopoietic cells. **F)** UMAP of PURE-seq sequenced patient PBMCs integrated with publicly available healthy PBMCs identifies 7 clusters, including 3 T/NK cells clusters, 2 monocytes clusters, 1 dendritic cells cluster, and 1 CTC-enriched cluster (dotted line). **G)** UMAP by data source of 10X PBMCs, PURE-seq ctrl (CD45+) cells, and PURE-seq neg (CD45-) cells. **H)** CTCs identified from PURE-seq enriched samples are highlighted in red.

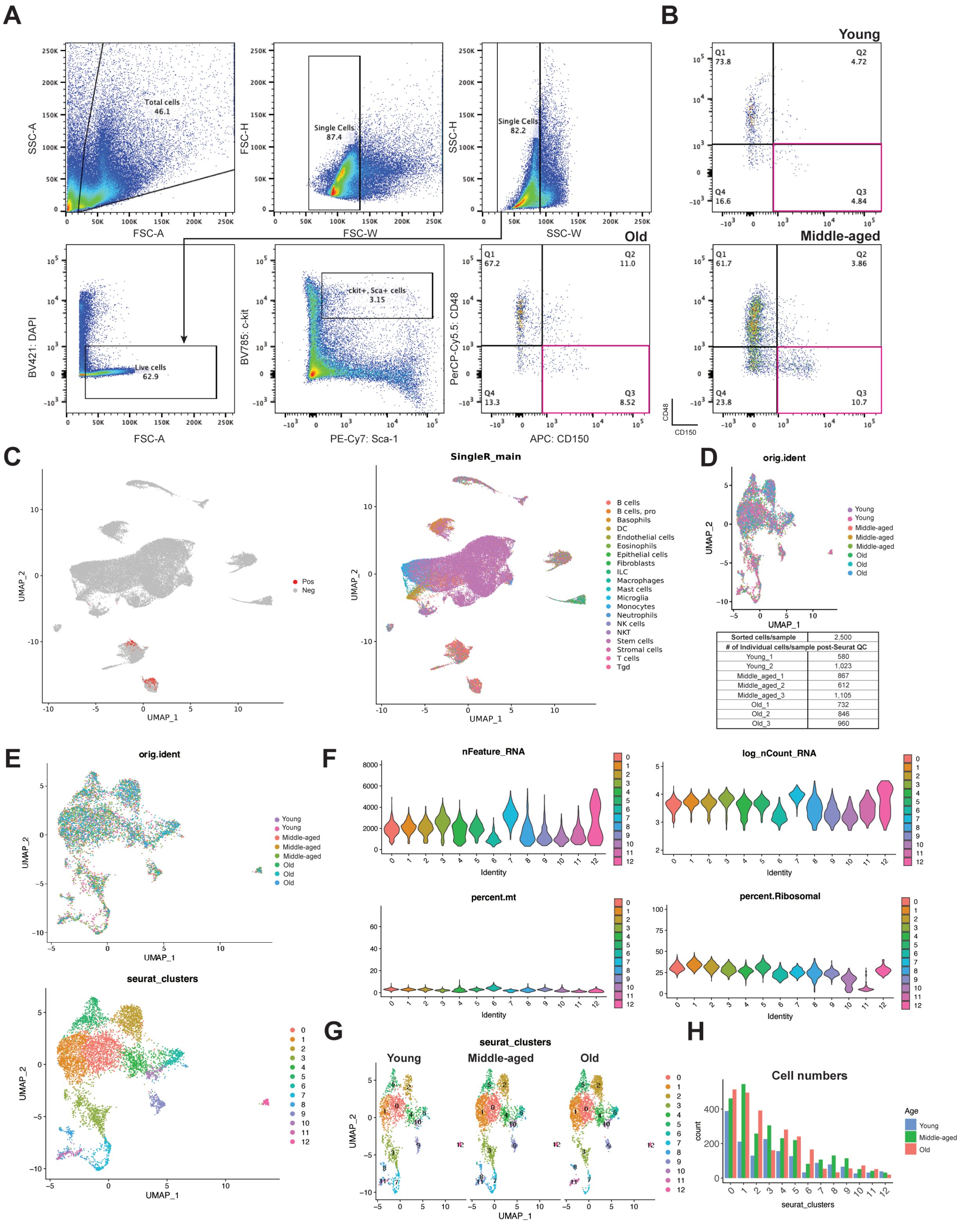


| Sorted cells/sample | 2,500 |
|---|---|
| # of Individual cells/sample post-Seurat QC | |
| Young_1 | 580 |
| Young_2 | 1,023 |
| Middle_aged_1 | 867 |
| Middle_aged_2 | 612 |
| Middle_aged_3 | 1,105 |
| Old_1 | 732 |
| Old_2 | 846 |
| Old_3 | 960 |

**Figure S3. Sorting of murine long-term repopulating hematopoietic stem cell and quality control analysis A)** Representative FACS plots using the gating strategy to sort LT-HSCs using old cells as an example. **B)** Representative FACS plots for young (top) and middle-aged

(bottom) LT-HSCs. **C)** UMAP plots of pre-sort samples, indicating LT-HSCs as labeled by scGate (left) and unbiased clustering by cell type using the SingleR package[59] (right). **D)** Integrated UMAP plot of samples from young (n=2), middle-aged (n=3), and old (n=3) mice (top) and the number of sorted cells per sample (n=2,500) and the number of cells recovered after passing quality control standards using the Seurat v4 pipeline, totaling 6,725 cells. **E)** Larger view of the integrated UMAP plot of samples from young (n=2), middle-aged (n=3), and old (n=3) samples, with each age group combining 4-6 mice. Colors indicate the age of the source mice (top) and the clustering of the 6,725 cells using the Seurat v4 pipeline (bottom). **F)** The number of unique genes (nFeature RNA), transcripts (nCount RNA as a logarithmic value), percent mitochondrial reads (percent.mt), and percent ribosomal reads (percent. Ribosomal) as a function of the cluster. **G)** Seurat clustering of young, middle-aged, and old samples. **H)** Bar graph illustrating the cell count for each age group within each Seurat cluster.

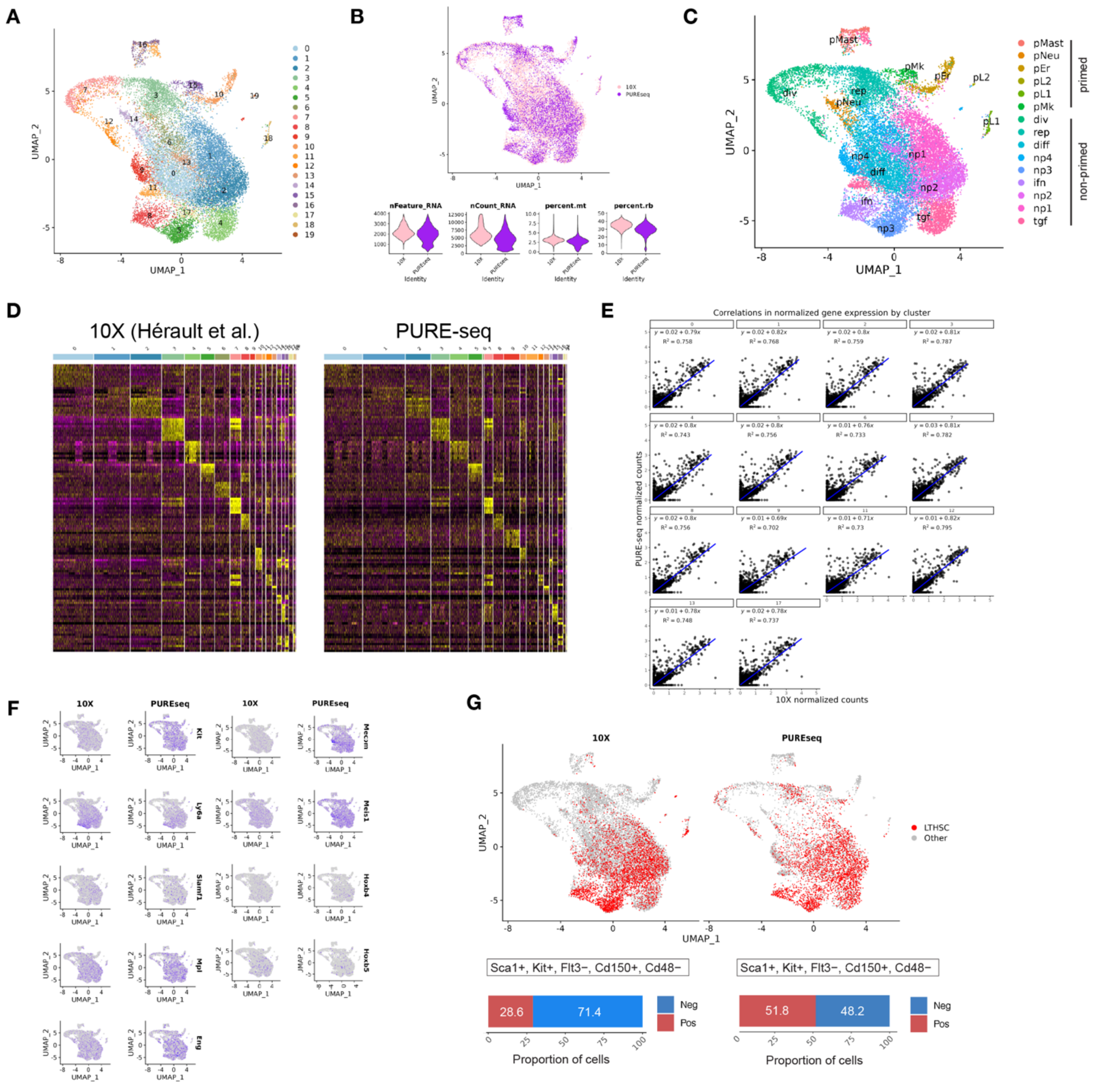


**Figure S4. Comparison of PURE-seq data to 10X Genomics data from Héuralt *et al*.[36] A)** UMAP plot of integrated PURE-seq and 10X Genomics dataset. **B)** UMAP plot by dataset and violin plots of qc features. **C)** UMAP plot with cell type annotation by scType. **D)** Heat maps of marker gene expression show similar patterns in PURE-seq and 10X data. **E)** Correlations in normalized gene expression by cluster between the datasets. **F)** UMAP plot showing expression of LT-HSC marker genes is consistent between the datasets. **G**) scGate analysis shows PURE-seq has a higher proportion of LT-HSCs compared to 10X dataset.

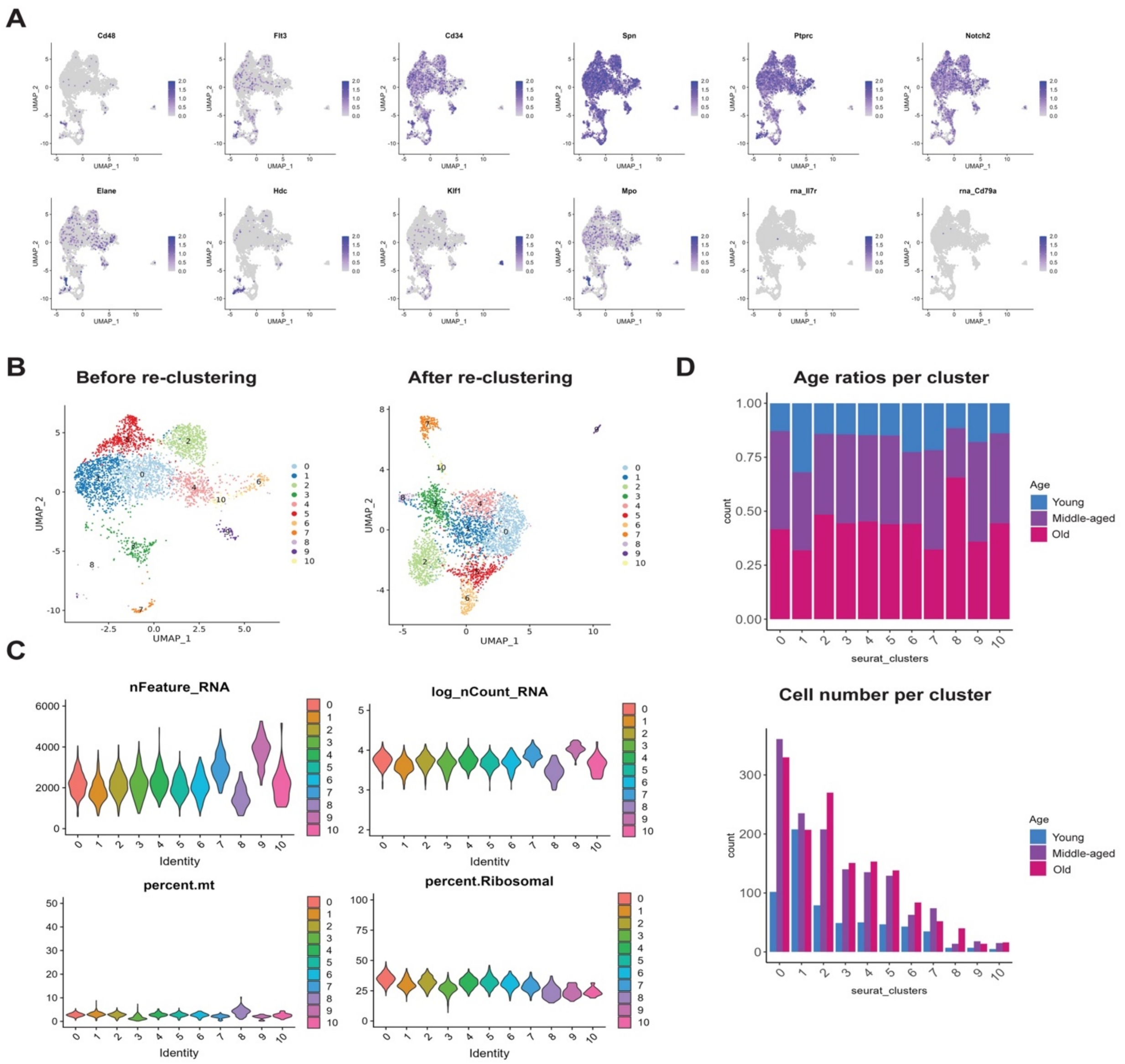


**Figure S5. UMAP analysis and clustering of Sca1⁺Kit⁺Flt3⁻CD150⁺CD48⁻ HSPCs for quality control and quantification. A)** UMAP plots showing expression of selected markers, including undifferentiated HSPC markers (e.g., Procr, Notch2) and lineage-associated markers (e.g., Mpo, Il7r). **B)** UMAP plots of Sca1⁺Kit⁺Flt3⁻CD150⁺CD48⁻ HSPCs before (left) and after (right) re-clustering. **C)** Quality control metrics per cluster following re-clustering: number of detected genes (nFeature_RNA), transcript counts (log-transformed nCount_RNA), percentage of mitochondrial reads (percent.mt), and percentage of ribosomal reads (percent.ribosomal). **D)** Bar graphs showing the proportion (top) and absolute number (bottom) of cells from each age group within each Seurat cluster after re-clustering.

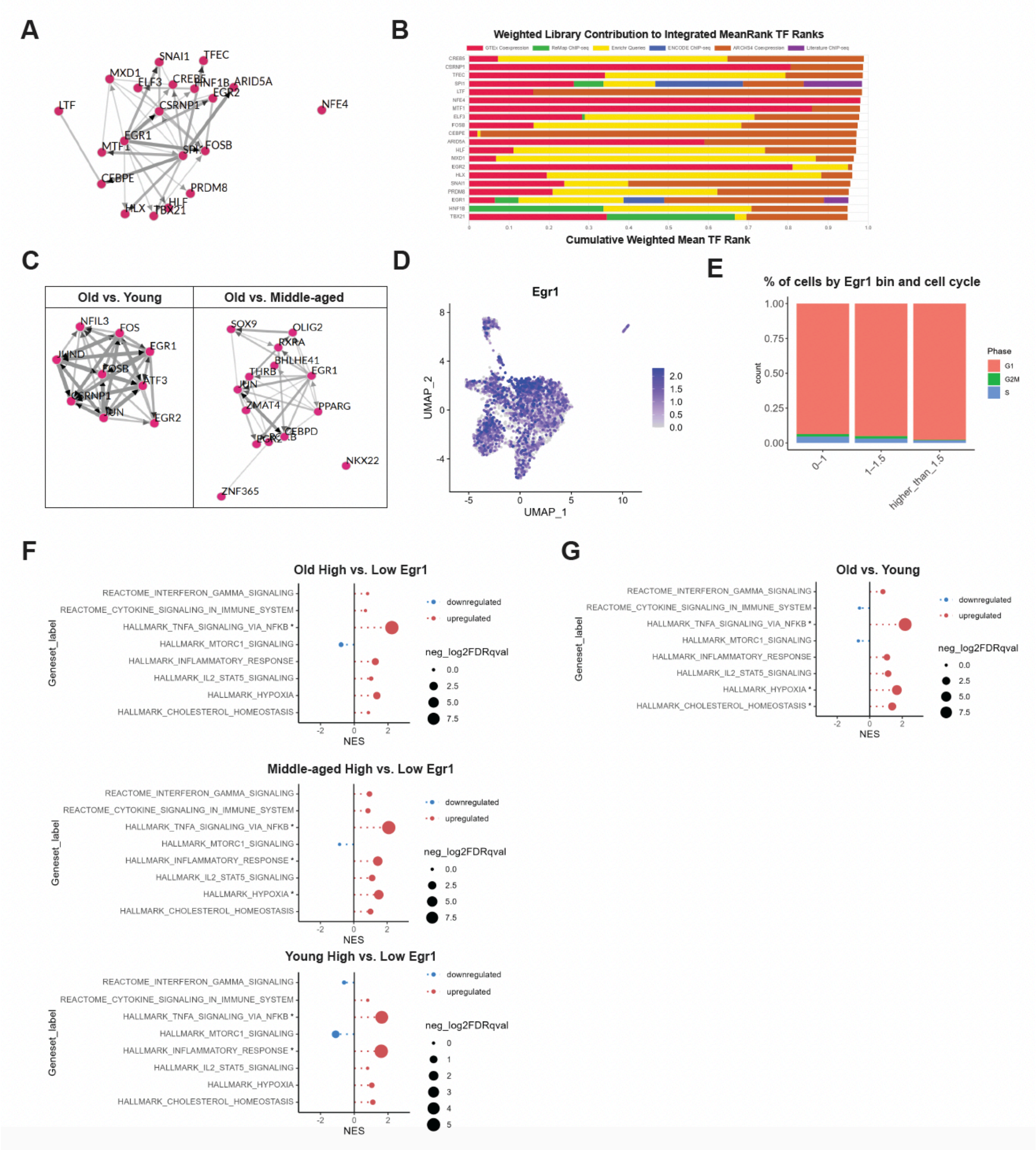


**Figure S6 ChEA3 and GSEA analyses identify *Egr1* as a top-ranked transcription factor driving gene upregulation in aging Sca1$^+$Kit$^+$Flt3$^-$CD150$^+$CD48$^-$ HSPCs, associated with increased inflammatory gene expression. A)** ChEA3-derived transcription factor (TF) network underlying DEGs between young and old LT-HSCs from Young et al. (2022). **B)** Ranking of TFs identified in A). **C)** ChEA3 TF networks comparing upregulated genes in old versus young (left) and old versus middle-aged (right) Sca1$^+$Kit$^+$Flt3$^-$CD150$^+$CD48$^-$ HSPCs. **D)** UMAP plot integrating Sca1$^+$Kit$^+$Flt3$^-$CD150$^+$CD48$^-$ HSPCs from young, middle-aged, and old mice. **E)** Bar graphs showing proportions of *Egr1*-expressing cells across cell cycle phases, stratified by *Egr1*

expression levels binned into low (0–1), mid (1–1.5), and high (>1.5). **F)** GSEA comparing old (top), middle-aged (middle), and young (bottom) *Egr1*high versus *Egr1*low $Sca1^{+}Kit^{+}Flt3^{-}CD150^{+}CD48^{-}$ HSCs. **G)** GSEA results comparing old versus young $Sca1^{+}Kit^{+}Flt3^{-}CD150^{+}CD48^{-}$ HSCs.
