# Supplementary material for: PURE-seq integrates FACS and PIP-seq for single-cell genomics of ultra-rare cells
Source: Nat Commun. 2026 Jan 21;17:1408. doi: 10.1038/s41467-025-68146-w (PMC12881479; doi:10.1038/s41467-025-68146-w)
Supplement: Supplementary file 2 — Description of Additional Supplementary Files [file 41467_2025_68146_MOESM2_ESM.pdf]

Description of Additional Supplementary Files

File name: Supplementary Data 1

Description: Cluster marker gene list of cells after PURE-seq enrichment of CTCs from peripheral blood samples of patients with metastatic melanoma (n=2).

File name: Supplementary Data 2

Description: Enrichr result on Jensen DISEASES for cluster 1 CTCs.

File name: Supplementary Data 3

Description: Enrichr result on Jensen DISEASES for cluster 2 CTCs.

File name: Supplementary Data 4

Description: DEGs CTCs vs hematopoietic cells from PURE-seq.

File name: Supplementary Data 5

Description: DEGs cluster 1 CTCs vs hematopoietic cells from PURE-seq.

File name: Supplementary Data 6

Description: DEGs cluster 2 CTCs vs hematopoietic cells from PURE-seq.

File name: Supplementary Data 7

Description: Gene set enrichment analysis with HALLMARK dataset on DEGs from cluster 1 CTCs vs HPCs.

File name: Supplementary Data 8

Description: Gene set enrichment analysis with HALLMARK dataset on DEGs from cluster 2 CTCs vs HPCs.

File name: Supplementary Data 9

Description: Cluster marker gene list for hematopoietic stem and progenitor cells (HSPCs) after PURE-seq enrichment of LT-HSCs from young (n=2), middle-aged (n=3), and old (n=3) mice samples.

File name: Supplementary Data 10

Description: Cluster marker gene list for HSPCs from integrated PURE-seq and Hérault et al. 10x dataset.

File name: Supplementary Data 11

Description: scType cell annotation of integrated PURE-seq and 10x HPSCs.

File name: Supplementary Data 12

Description: LT-HSCs identification using scGate analysis for PURE-seq enriched HSPCs.
