# Supplementary material for: PURE-seq integrates FACS and PIP-seq for single-cell genomics of ultra-rare cells
Source: Nat Commun. 2026 Jan 21;17:1408. doi: 10.1038/s41467-025-68146-w (PMC12881479; doi:10.1038/s41467-025-68146-w)
Supplement: Supplementary file 4 — Reporting Summary [file 41467_2025_68146_MOESM4_ESM.pdf]

nature portfolio

Corresponding author(s): Adam Abate
Ross Levine

Last updated by author(s): Oct 30, 2025

Reporting Summary

Nature Portfolio wishes to improve the reproducibility of the work that we publish. This form provides structure for consistency and transparency in reporting. For further information on Nature Portfolio policies, see our Editorial Policies and the Editorial Policy Checklist.

Statistics

For all statistical analyses, confirm that the following items are present in the figure legend, table legend, main text, or Methods section.

n/a	Confirmed	
☐	☒	The exact sample size (*n*) for each experimental group/condition, given as a discrete number and unit of measurement
☐	☒	A statement on whether measurements were taken from distinct samples or whether the same sample was measured repeatedly
☐	☒	The statistical test(s) used AND whether they are one- or two-sided<br>*Only common tests should be described solely by name; describe more complex techniques in the Methods section.*
☒	☐	A description of all covariates tested
☐	☒	A description of any assumptions or corrections, such as tests of normality and adjustment for multiple comparisons
☐	☒	A full description of the statistical parameters including central tendency (e.g. means) or other basic estimates (e.g. regression coefficient) AND variation (e.g. standard deviation) or associated estimates of uncertainty (e.g. confidence intervals)
☐	☒	For null hypothesis testing, the test statistic (e.g. F, t, r) with confidence intervals, effect sizes, degrees of freedom and P value noted<br>*Give P values as exact values whenever suitable.*
☒	☐	For Bayesian analysis, information on the choice of priors and Markov chain Monte Carlo settings
☒	☐	For hierarchical and complex designs, identification of the appropriate level for tests and full reporting of outcomes
☒	☐	Estimates of effect sizes (e.g. Cohen's *d*, Pearson's *r*), indicating how they were calculated

Our web collection on statistics for biologists contains articles on many of the points above.

Software and code

Policy information about availability of computer code

Data collection	No custom software or code were used for data collection
Data analysis	The open-source software, tools, and packages used for data analysis in this study, as well as the version of each program, were R (v3.6.1), PIPseeker (v1.0.0), Seurat R package (v4.3.0), scGate R package (v1.6), ScType R package (v1.0), SingleR R package (v1.0). No custom software, tools, or packages were used.

For manuscripts utilizing custom algorithms or software that are central to the research but not yet described in published literature, software must be made available to editors and reviewers. We strongly encourage code deposition in a community repository (e.g. GitHub). See the Nature Portfolio guidelines for submitting code & software for further information.

Data

Policy information about availability of data

All manuscripts must include a data availability statement. This statement should provide the following information, where applicable:

- Accession codes, unique identifiers, or web links for publicly available datasets
- A description of any restrictions on data availability
- For clinical datasets or third party data, please ensure that the statement adheres to our policy

Sequencing data were deposited into the NCBI Gene Expression Omnibus under GSE273803.

Research involving human participants, their data, or biological material

Policy information about studies with human participants or human data. See also policy information about sex, gender (identity/presentation), and sexual orientation and race, ethnicity and racism.

Reporting on sex and gender	N/A, information not collected
Reporting on race, ethnicity, or other socially relevant groupings	N/A, information not collected
Population characteristics	N/A, information not collected
Recruitment	As part of routine clinical practice at UCSF, all patients included in this study signed an informed consent waiver to contribute de-identified data to scientific research projects.
Ethics oversight	UCSF Institutional Review Board (22-37134)

Note that full information on the approval of the study protocol must also be provided in the manuscript.

Field-specific reporting

Please select the one below that is the best fit for your research. If you are not sure, read the appropriate sections before making your selection.

☒ Life sciences ☐ Behavioural & social sciences ☐ Ecological, evolutionary & environmental sciences

For a reference copy of the document with all sections, see nature.com/documents/nr-reporting-summary-flat.pdf

Life sciences study design

All studies must disclose on these points even when the disclosure is negative.

Sample size	Sample size calculation was not performed but rather, all experiments were performed in biological duplicate or triplicate to ensure reproducibility. Sample numbers were chosen based on prior studies using comparable single-cell sequencing approaches and were sufficient to capture biological variability and yield robust clustering and differential expression results.
Data exclusions	No data was excluded
Replication	All experiments were performed with a minimum of biological duplicate
Randomization	Human data was obtained prospectively and randomization was not performed.
Blinding	Investigators were not blinded to group allocation during data collection or analysis. Blinding was not relevant to this study because cell population groups were objectively defined by flow-cytometric gating and computational clustering of single-cell transcriptomic data.

Reporting for specific materials, systems and methods

We require information from authors about some types of materials, experimental systems and methods used in many studies. Here, indicate whether each material, system or method listed is relevant to your study. If you are not sure if a list item applies to your research, read the appropriate section before selecting a response.

Materials & experimental systems

n/a	Involved in the study	
☐	☒	Antibodies
☐	☒	Eukaryotic cell lines
☒	☐	Palaeontology and archaeology
☐	☒	Animals and other organisms
☐	☒	Clinical data
☒	☐	Dual use research of concern
☒	☐	Plants

Methods

n/a	Involved in the study	
☒	☐	ChIP-seq
☐	☒	Flow cytometry
☒	☐	MRI-based neuroimaging

Antibodies

Antibodies used	The cells used in mouse-human mixing experiment were stained with: The target population was treated with 1 µg/mL Calcein Red-Orange (Invitrogen, C34851), and the background population was treated with 1 µg/mL Calcein Green (Invitrogen, C34852). The patient-derived PBMCs were then stained with human TruStain FcX (Biolegend, 422301), APC anti-human CD45 (Biolegend, 304011), and Calcein AM (Biolegend, 425201). To label mice LT-HSC cells, the following fluorophore-conjugated antibodies were used at the

	indicated dilutions: CD117 (c-Kit) BV785 (clone 2B8, BioLegend; 1:200 dilution), Ly-6A/E (Sca-1) PE/Cy7 (clone D7, BioLegend; 1:1000 dilution), CD48 PerCP/Cy5.5 (clone HM48-1, BioLegend; 1:100 dilution) and CD150 (SLAM) APC (clone TC15-12F12.2, BioLegend; 1:50 dilution).
Validation	Antibodies validated by manufacturers for all reagents

Eukaryotic cell lines

Policy information about cell lines and Sex and Gender in Research

Cell line source(s)	Cell lines used include HEK-293T, NIH 3T3 cells (ATCC).
Authentication	Cell cultures were authenticated by short tandem repeat (STR) analysis at the UC Berkeley DNA Sequencing Facility
Mycoplasma contamination	All cell lines were tested for mycoplasma and found negative. Our research group carries out mycoplasma testing once per month on all active cell lines.
Commonly misidentified lines (See ICLAC register)	None

Animals and other research organisms

Policy information about studies involving animals; ARRIVE guidelines recommended for reporting animal research, and Sex and Gender in Research

Laboratory animals	young (2-3 months old), middle-aged (12-14 months old), and old (18-20 months old) female C57BL/6 mice
Wild animals	No wild animals were used in this study
Reporting on sex	Only female mice were used
Field-collected samples	No field collected samples were used in this study
Ethics oversight	Memorial Sloan Kettering Cancer Center under the Institutional Animal Care and Use Committee-approved animal protocol (#07-10-016)

Note that full information on the approval of the study protocol must also be provided in the manuscript.

Clinical data

Policy information about clinical studies

All manuscripts should comply with the ICMJE guidelines for publication of clinical research and a completed CONSORT checklist must be included with all submissions.

Clinical trial registration	not applicable
Study protocol	*Note where the full trial protocol can be accessed OR if not available, explain why.*
Data collection	*Describe the settings and locales of data collection, noting the time periods of recruitment and data collection.*
Outcomes	*Describe how you pre-defined primary and secondary outcome measures and how you assessed these measures.*

Plants

Seed stocks	not applicable
Novel plant genotypes	*Describe the methods by which all novel plant genotypes were produced. This includes those generated by transgenic approaches, gene editing, chemical/radiation-based mutagenesis and hybridization. For transgenic lines, describe the transformation method, the number of independent lines analyzed and the generation upon which experiments were performed. For gene-edited lines, describe the editor used, the endogenous sequence targeted for editing, the targeting guide RNA sequence (if applicable) and how the editor was applied.*
Authentication	*Describe any authentication procedures for each seed stock used or novel genotype generated. Describe any experiments used to assess the effect of a mutation and, where applicable, how potential secondary effects (e.g. second site T-DNA insertions, mosiacism, off-target gene editing) were examined.*

Flow Cytometry

Plots

Confirm that:

- [x] The axis labels state the marker and fluorochrome used (e.g. CD4-FITC).
- [x] The axis scales are clearly visible. Include numbers along axes only for bottom left plot of group (a 'group' is an analysis of identical markers).
- [x] All plots are contour plots with outliers or pseudocolor plots.
- [x] A numerical value for number of cells or percentage (with statistics) is provided.

Methodology

Sample preparation	We harvested whole BM cells from young (2-3 months old), middle-aged (12-14 months old), and old (18-20 months old) C57BL/6 mice. To enrich for HSPCs, lineage-positive cells were depleted using magnetic-activated cell sorting (MACS), after which Sca1^{+}Kit^{+}CD150^{+}CD48^{-} cells were sorted from the pooled BM samples (n = 2–3 mice per pool) using the PURE-seq workflow.
Instrument	BD FACS Aria III, BD FACSymphony™ S6
Software	BD FACS
Cell population abundance	To robustly identify the target cell population, we applied scGate29, a marker-based purification algorithm previously benchmarked on scRNA-seq datasets that efficiently isolates defined cell populations of interest. Our analysis revealed that 51.8% of captured cells were Sca1^{+}Kit^{+}Flt3^{-}Slamf1^{+}CD48^{-}
Gating strategy	we sorted from Lineage−Sca-1+c-Kit+ (LSK) cells based on the expression of SLAM markers to enrich for CD150+CD48− LSK cells

- [x] Tick this box to confirm that a figure exemplifying the gating strategy is provided in the Supplementary Information.
